# The impact of Emotion-focused training for emotion couching delivered as mobile app on self-compassion and self-criticism

**DOI:** 10.3389/fpsyg.2022.1047022

**Published:** 2023-01-24

**Authors:** Júlia Halamová, Jakub Mihaľo, Lukáš Bakoš

**Affiliations:** Faculty of Social and Economic Sciences, Institute of Applied Psychology, Comenius University in Bratislava, Bratislava, Slovakia

**Keywords:** self-compassion, self-criticism, self-protection, emotion, emotion focused therapy, mobile app, mHealth, MHapps

## Abstract

**Introduction:**

Being self-compassionate is considered a beneficial emotion regulation strategy. Therefore, the acquisition of emotional skills can raise self-compassion levels and consequently reduce self-criticism.

**Methods:**

Hence, the goal of the current study was to develop a mobile app based on the empirically proven group version of Emotion-Focused Training for Emotional Coaching (EFT-EC) and test its effectiveness in reducing self-criticism and raising self-compassion and self-protection. The sample consisted of 85 participants, of whom 22.4% were men and 77.6% were women. The mean age was 32.53 (SD = 14.51), ranging from 18 to 74 years. The participants filled out the following scales immediately before and after using the fourteen-day mobile app: The Forms of Self-Criticizing/Attacking & Self-Reassuring Scale (FSCRS), The Sussex-Oxford Compassion for the Self Scale (SOCS-S), and The Short-form Version of The Scale for interpersonal behaviour (s-SIB).

**Results:**

Use of the 14-day EFT-EC mobile app significantly improved self-compassion and self-reassurance and significantly reduced self-criticism compared to pre- and post-measurements.

**Discussion:**

The results are promising as self-criticism is a transdiagnostic phenomenon observed in various kinds of psychopathology and reducing it may prevent the emergence of psychopathologies. Moreover, the mobile app intervention can easily be accessed by a wide range of users, without requiring the services of a mental health professional, and thereby reduces the potential risk of shame or stigmatization.

## Introduction

According to Blatt and Zuroff (1992), self-criticism is constant, harsh self-scrutiny, in which the individual experiences negative emotions accompanied by feelings of unworthiness, shame, inferiority, and guilt. Elevated levels of self-criticism can lead to unhealthy perfectionism (Cox et al., 2002), shame (Gilbert and Irons, 2005), social anxiety, or post-traumatic stress disorder, and in extreme cases may cause suicidal ideations (O’Connor and Noyce, 2008). In addition, self-criticism has been linked to schizophrenia, depression, and borderline personality disorder (Blatt, 1974; Bergner, 1995). The opposite of self-criticism is self-compassion. Strauss et al. (2016) define compassion as a cognitive, affective, and behavioural process consisting of five elements: (1) recognition of suffering; (2) understanding the universality of suffering in human experience; (3) feeling empathy for the suffering person and compassion for his/her suffering (emotional resonance); (4) tolerating the discomfort elicited in response to the suffering person (e.g., restlessness, anger, fear) as well as the ability to remain open to and accept the suffering person; and (5) motivation to act to alleviate another person’s suffering. Self-compassion is compassion directed at oneself in situations of personal failure, or difficult situations, and reacting with understanding and kindness toward oneself (Neff, 2003).

Self-compassion and self-criticism have an enormous effect on mental health. Traditionally self-compassionate and self-critical interventions have been carried out in face-to-face individual or group therapy sessions. Kirby et al. (2017) in their metanalysis of interventions aimed at cultivating compassion toward oneself and others found they were effective in raising self-compassion, mindfulness, and well-being and reducing depression, anxiety, and psychological distress. MacBeth and Gumley (2012) found that self-compassionate interventions reduce symptoms of depression as well as anxiety and stress. Although traditional therapy methods are effective, they are expensive, time-consuming, and geographically bound (Chandrashekar, 2018). Technological advancements have been crucial to the development of more accessible and affordable psychological interventions. For example, in an online survey Wahbeh et al. (2014) discovered that participants significantly preferred internet-delivered mindfulness meditation interventions over group and individual in-person sessions.

However, online interventions have limited accessibility too. Participants are required to use a computer, laptop, or tablet, which may not always be available. Online interventions can be accessed through a smartphone, but these online websites are often not adapted for mobile use. Mobile mental health apps (MHapps) address all of these issues (Chandrashekar, 2018). Moreover, the number of smartphone users worldwide is expected to reach 6.6 billion in 2022 and to continue growing rapidly in subsequent years (Statista, 2021). MHapps are accessible, affordable, private, and do not require the person to be at a specific location (Chandrashekar, 2018).

The trade-off with MHapps is that they are not as personalized as therapist-delivered interventions (Bakker et al., 2016). This could be partly solved by creating an environment that is individually tailored and customized to needs. Furthermore, researchers (Anthes, 2016) have raised safety issues as MHapps have not been supported by academic research and therefore there is no efficiency research or even no harm research. Anyone can create an app without the need to comply with international guidelines and rules, as these do not exist yet (Anthes, 2016). Apps that are not backed by scientific data could potentially do psychological harm to individuals (Anthes, 2016). That is why it is essential to create scientifically tested mobile apps that are affordable and that could aid a large population of people without access to in-person psychotherapy.

### Emotion-focused therapy

Empirically supported methods for treating excessive self-criticism and low levels of self-compassion are emerging from the findings of Emotion-focused Therapy (EFT; Greenberg, 2011) and other related psychotherapy fields (e.g., Gilbert, 2009). According to EFT, emotions are a means of obtaining a better understanding of oneself and one’s needs. Not understanding emotions and/or avoiding unpleasant ones could cause harm and lead to psychological problems (Greenberg, 2011). EFT therapists guide clients through situations to increase emotional awareness, adaptation, and regulation, and to help them transform maladaptive emotions into adaptive emotions, make better use of adaptive emotions, and cope better with maladaptive emotions. The main transformative healing process in EFT requires the evocation of self-compassion and self-protection in order to combat self-criticism (Pascual-Leone and Greenberg, 2007).

Empirical research is increasingly focused on self-compassion and self-criticism in individuals because self-compassion and self-criticism are important aspects of well-being and countering psychopathology (Ferrari et al., 2019). Research shows that EFT is an effective method for increasing self-compassion and reducing self-criticism in the non-clinical population in the prevention of mental disorders (Halamová et al., 2021). EFT therapists aim to access primary adaptive emotions because these provide useful information for tackling the situation and for meeting the needs of the individual. To counter self-criticism individuals need both to increase their self-compassion and to deploy protective anger as that encourages them to stand up for themselves (Timulak, 2015; Vrana and Greenberg, 2018).

Protective anger is an effective response to maltreatment and unmet needs (Timulak, 2015). Protective anger helps the person to gain a sense of personal power, which helps them to face painful feelings (loneliness, shame, fear, etc.) rather than avoiding them and that enables them to transform maladaptive emotions into adaptive emotions under specific guidance and facilitation. In EFT, the more often feelings of self-compassion and protective anger are incorporated into the therapy, the more emotionally resilient and flexible the individual becomes (Timulak, 2015). It is our view that protective anger is closely related to, if not overlapping with assertiveness. Assertive people master abilities such as standing up for themselves without experiencing strong anxiety or clearly and directly expressing and verbalizing their feelings, thoughts, opinions, desires, and requirements without recourse to aggression and while respecting the rights of others (Speed et al., 2018). Assertive people have the ability not only to analyse their emotions, including the ability to clearly define their feelings, but also the ability to control their impulses and express their needs appropriately and proportionately (Stein and Book, 2006). Low levels of assertiveness can manifest in submissiveness, excessive aggression, and grumpiness toward others (Speed et al., 2018), and have been linked to depressive symptoms, social anxiety, life satisfaction, and other negative consequences (Peneva and Mavrodiev, 2013; Speed et al., 2018).

### Interventions research

These concepts self-criticism, self-protection, and self-compassion are an essential part of emotional training and are key elements of resolving emotional imbalance (Heffernan et al., 2010). Currently, several interventions target these issues. Compassion Mind Training, which is based on Compassion-focused Therapy (CFT; Gilbert, 2009), aims to enhance psychological and emotional healing through understanding and feeling compassion for oneself during negative thought processes, while focusing on nourishing compassion within the self. CFT helps individuals with a highly self-critical ‘inner voice’ develop a more compassionate point of view in situations of suffering. An early systematic review of CFT effectiveness concluded that this therapeutic method is effective in reducing high levels of self-criticism (Leaviss and Uttley, 2014). Another intervention dealing with emotion is Cultivating Emotional Balance (CEB; Kemeny et al., 2012), which uses emotional training to help individuals experience and express emotions toward themselves and others and reduce negative emotional states. CEB integrates scientific knowledge (understanding of emotions, emotion triggers, experience, and consequences), Eastern contemplative practices, and philosophy of emotion. Participants undertake 42 h of topic presentations, group and pair discussions, guided practice exercises in meditation, and emotion regulation training. Empirical findings suggest that CEB may help individuals improve the regulation of emotions, and enhance mindfulness, self-care, and self-compassion capabilities (Kemeny et al., 2012; Sansó et al., 2017).

As stated previously, these interventions are almost exclusively delivered through face-to-face sessions (Gilbert, 2009; Greenberg, 2011; Kemeny et al., 2012). However, recently a couple of papers have explored the use of technologies (mostly online interventions) in self-compassionate training. Finlay-Jones et al. (2016) created an online platform focused on individual self-compassionate intervention. Participants were psychology students, and the intervention had a significant positive effect, increasing self-compassion and happiness, and reducing depressiveness, distress, and emotional regulation. Another study (Krieger et al., 2016) deployed an online self-compassion-based intervention among people with excessive self-criticism. Researchers found a significant long-term (3.5 months) reduction in self-criticism, stress, fear of self-compassion, and elevated self-compassion, life satisfaction, and mindfulness. Similarly, Eriksson et al. (2018) tested the effectiveness of online mindfulness-compassion training on stress and professional burnout amongst practicing psychologists. They measured outcomes in self-compassion and mindfulness, self-coldness, stress, and professional burnout. The online web intervention had a significant positive effect on all of these. Recently, research teams have attempted to create empirically supported MHapps for self-compassion. Mak et al. (2018) concluded that mindfulness, self-compassionate, and cognitive behavioural psychoeducation delivered through a mobile app was appropriate for improving mental well-being and reducing stress over the long-term. Linardon et al. (2019) reviewed the recent literature and in his meta-analytic review concluded that self-compassion and mindfulness could be elevated through MHapps. Orosa-Duarte et al. (2021) compared the effect of the mindfulness MHapp versus face-to-face mindfulness intervention among a student healthcare population. The mobile app group outperformed the in-person intervention group in terms of reduced anxiety. Both intervention groups reported a significant increase in self-compassion and mindfulness.

Elevated levels of self-compassion and lower self-criticism correlate with higher scores on happiness and well-being scales (Zessin et al., 2015). According to Gilbert and Irons (2004), self-criticism can be treated by cultivating compassion and self-compassion. Furthermore, Kemeny et al. (2012) proposed that the acquisition of emotional skills can raise levels of compassion and self-compassion. This was further supported by Heffernan et al. (2010), who confirmed a relationship between emotional intelligence and self-compassion. Moreover Beaumont et al. (2016) discovered a relation between self-compassion and emotional resilience. The protective role of self-compassion helps reduce chronic stress and its effect on emotional responses (Neff and Vonk, 2009). Neff (2003) considers self-compassion to be a beneficial emotional regulation strategy. That is, negative emotions, psychological distress, and painful feelings are accepted through kindness, understanding, and a non-judgmental attitude. Maslow (1997) argued that emotional maturity is associated with non-judgmental being, forgiveness, and acceptance of oneself as well as others. This is further supported by Neff and Vonk, 2009, who claimed that self-compassion is strongly linked to emotional intelligence and wisdom. In addition, Lazarus and Folkman (1984) have argued that people high in compassion often adopt emotional and adaptive coping responses to stress. Furthermore, self-compassion is positively linked with emotionally focused adaptive coping strategies and negatively associated with maladaptive coping responses in those faced with failure (Lazarus and Folkman, 1984). Similarly, a sample of participants with high self-compassion were more likely to transform negative emotional states into positive ones, which results in action and appropriate adaption and change (Carver and Connor-Smith, 2010). Hence, we wanted to know whether cultivating emotional intelligence skills would have an impact on self-criticism and self-compassion (Halamová and Kanovský, 2019) and possibly self-protection.

As there was no existing mobile app focusing on the emotional aspects of individual mental health, we decided to create an app empirically supported by the latest EFT research findings and delivered through an affordable and available intervention targeting a negative aspect of high self-criticism and low self-compassion. We therefore selected an intervention by Halamová and Kanovský (2019), who created the Emotion-Focused Training for Emotional Coaching (EFT-EC), which integrates modules on emotional intelligence, self-compassion, and self-criticism. The training was developed using up-to-date knowledge on Emotion-Focused Therapy and previous empirical research and consists of 14 individual home exercises, as well as groups sessions, discussions, and group activities. The results showed a significant effect on participants’ self-criticism and self-control levels. We decided to create a mobile app that would provide an affordable, empirically supported, and easy-to-access online intervention for emotional training.

## The aim of the research study

Hence, our goal was to develop a mobile app of the original group version of the empirically supported Emotion-Focused Training for Emotional Coaching (EFT-EC; Halamová and Kanovský, 2019) and test its effectiveness in reducing self-criticism and raising self-compassion and self-protection. Based on this, our hypotheses were:

1. the EFT-EC intervention will significantly raise participants’ self-compassion.
2. the EFT-EC intervention will significantly reduce participants’ self-criticism.
3. the EFT-EC intervention will significantly raise participants’ self-protection/assertiveness.

## Methods

### The research sample

The sample consisted of 85 Slovak-speaking participants (82 Slovak, 2 Czech, 1 Hungarian), of whom 22.4% were men and 77.6% were women. Mean age was 32.53 (SD = 14.51), ranging from 18 to 74 years. In total our sample contained 218 participants who had registered for the app. The drop-out rate was 61.0%. Regarding the 133 incomplete interventions, most participants did not finish any of the tasks (39.7%), 16.5% of participants dropped out on the second day, 20.3% on the third day, 3.8% on the fourth day, 0% on the fifth day, 6.8% on the sixth day, 0.8% on the seventh day, 3.8% on the eight day, 3% on the nineth day, 1.5% on the tenth day, 0% on the eleventh day, 0% on the twelfth day, 0 the thirteenth day, and 0% on the last day. Furthermore 3.8% participants completed the whole intervention and all exercises but did not complete the intervention questionnaires at post-measurement. Presented drop-out rates are slightly higher than other mental health studies leveraging mobile apps to distribute interventions (Torous et al., 2020) and comparable to internet interventions of Mellor et al. (2008). All participants completed an online informed consent form. Data were collected in accordance with the ethical standards of the institutional and/or national research committee and following the 1964 Helsinki Declaration and its later amendments or comparable ethical standards. The study protocol was approved by the ethical committee of a related university.

### Measurement instruments

#### The Forms of Self-Criticizing/Attacking and Self-Reassuring Scale

The Forms of Self-Criticizing/Attacking and Self-Reassuring Scale (FSCRS) scale was developed by Gilbert et al. (2004) and will be used to measure self-criticism and self-reassurance among the mobile app participants. The scale consists of 22 items measuring three components: Reassured Self (RS), Inadequate Self (IS), and Hated Self (HS). Inadequate Self and Hated Self (IS+HS) measure personal inadequacy and the desire to hurt oneself, respectively, and together sum up to a single self-criticism score. Reassured Self focuses on being able to forgive oneself, which is similar to self-compassion. The items are rated on a 5-point Likert scale ranging from 0 (“Not at all like me”) to 4 (“Extremely like me”). Example scale items are: for Inadequate Self – *“I remember and dwell on my failings,”* for Reassured Self – *“I am gentle and supportive with myself,”* and for Hated Self – *“I have become so angry with myself that I want to hurt or injure myself.”* The internal consistency of the total FSCRS was excellent, *α* = 0.90 (Baião et al., 2014). The analysis of the subscales revealed values of 0.82 (Reassured Self), 0.86 (Inadequate Self), and 0.80 (Hated Self) (Baião et al., 2014). In addition, the FSCRS scale showed very good psychometric properties in terms of reliability, validity, and factor structure when tested on a Slovak population sample (Halamová et al., 2017). The analysis of the results for the Slovak population showed values of 0.85 (Inadequate Self), 0.75 (Hated Self), and 0.75 (Reassured Self) (Halamová et al., 2017).

#### The Sussex-Oxford Compassion for the Self Scale

The Sussex-Oxford Compassion for the Self Scale (SOCS-S) was developed by Gu et al. (2020) and was used in this study to measure participant levels of self-compassion. This scale is based on a theoretically and empirically supported definition of compassion comprising five dimensions: (a) recognizing suffering (RS), (b) understanding the universality of suffering (US), (c) feeling for the person suffering (FS), (d) tolerating uncomfortable feelings (TS), and (e) motivation to act/acting to alleviate suffering (MA). The items are rated on a 5-point Likert scale ranging from 1 (“Not at all true”) to 4 (“Always true”). Example scale items include: for Recognizing Suffering – *“I recognise signs of suffering in myself,”* for Understanding the Universality of Suffering – *“Like me, I know that other people also experience struggles in life,”* for Feeling for the Person Suffering – *“When I’m going through a difficult time, I feel kindly towards myself,”* for Tolerating Uncomfortable Feelings – *“I connect with my own suffering without judging myself,”* and for Motivation to Act/Acting to Alleviate Suffering – *“I try to make myself feel better when I’m distressed, even if I cannot do anything about the cause.”* The results of the psychometric analysis of the scale showed adequate internal consistency, interpretability, floor/ceiling effects, and convergent and discriminant validity (Gu et al., 2020). The SOCS scales were translated and tested on the Slovak population (Halamová and Kanovský, 2021). The results of the analysis showed good psychometric properties for both subscales (Halamová and Kanovský, 2021) – Compassion for Others (*α* = 0.93) and Compassion for the Self (*α* = 0.89).

#### The short version of the Scale for Interpersonal Behavior

The short-form version of the Scale for Interpersonal Behaviour (s-SIB; Arrindell et al., 2002) was developed to measure levels of Negative Assertion (standing up for oneself), Positive Assertion (ability to give and receive praise), Initiating Assertiveness (ability to socialize in everyday situations), and Expression of and Dealing with Personal Limitations (dealing with pressure, recognizing one’s failure, failures, and criticism). A total of 25 items are rated on a 5-point Likert scale on two different measures – Distress (s-SIB), which is the discomfort participants feel in the situation described in the particular item (not at all, somewhat, rather, very, and extremely) and Performance (s-SIBF), which the is frequency of the described behaviour (I never do, I rarely do, I sometimes do, I usually do, I always do). Example scale items include: for Negative Assertions (NA for distress, FNA for performance) – “Refusing to lend something to a near acquittance,” for Expression and Dealing with Personal Limitations – “Asking someone to show you the way,” for Initiation Assertiveness (IA for distress, FIA for performance) – “Giving your opinion to a person in authority,” and for Positive Assertions (PA for distress, FPA for performance) – “Telling someone that you like him/her.” The s-SIB scales were translated and tested on the Slovak population (Vráblová and Halamová, 2022). The results of the analysis showed good psychometric properties: Cronbach α coefficients for all subscales were – Positive Assertion (distress *α* = 0.93 and performance *α* = 0.94), Negative Assertion (distress *α* = 0.83 and performance *α* = 0.82), Initiating Assertiveness (distress *α* = 0.83 and performance *α* = 0.81), and Expression of and Dealing with Personal Limitations (distress *α* = 0.79 and performance *α* = 0.81). The results of the Mokken analysis supported using the total scores for s-SIB distress and s-SIB performance and their subscales.

#### EFT-EC mobile application

The participants of the EFT-EC intervention undertook a 14-day intervention, where the goal was to raise self-compassion and self-protection and reduce self-criticism. Each task began with a short psychoeducational section that provided basic information about the task at hand. All 14 tasks took approximately 15 min to complete. Participants were asked questions and had to reply using expressive writing to deepen the effect of the intervention (Pennebaker and Beall, 1986; Pennebaker, 2017). At the end of each task, we asked the following questions: How did you feel during the exercise? (emotional aspect). What did you realize during the exercise? (cognitive aspect). What can you take from the exercise for use in everyday life? (behavioural aspect). Participants could complete one task only within a 24-h period so as to give them time for reflection and to space out the tasks. This also served as a check on participants’ commitment to the intervention. The mobile app was internet based and did not work offline as it was necessary to collect data about the participants.

The EFT-EC intervention is focused on raising awareness of emotions and current behavioural patterns toward oneself and other people in everyday situations. Participants are taught effective and healthy ways to cope with and manage emotions and to enhance their emotional intelligence, which often results in better work performance and relationship satisfaction. Participants who completed the intervention could compare their pre and post-intervention scores using interactive graphs. The scores were calculated from the initial and post-intervention questionnaire responses. This provided participants with a motivational tool and a tangible result.

**Day 0: Initial measures**

Pre-measurements.

**Day 1: Examining my emotional closeness in relationships** (inspired by Gottman and Silver, 2000; Johnson, 2011; Halamová and Kanovský, 2019)

This exercise consists of questions exploring the participants’ attitudes to their emotions based on their upbringing, sociocultural background, and the way they form emotional closeness to loved ones or how comfortable they are with asking for closeness or other needs to be met in their relationships.

**Day 2: Self-soothing with sensory perception** [inspired by Halamová and Kanovský (2019), Linehan (1993), and Segal (2008)]

The goal of this exercise is to find ways to calm down using readily available tools such as using one’s senses to calm oneself down and induce a pleasant feeling. During this exercise, participants can explore a wide range of sensory sensations through motion, sight, hearing, touch, smell, and taste.

**Day 3: Safe space** [inspired by Greenberg and Warwar (2006) and Halamová and Kanovský (2019)]

Imagining a safe place is another way of preparing to cope with the potential stress of waves of feelings. Participants are instructed to imagine a safe space they can always return to. The purpose of this guided meditation is to teach participants how to calm themselves down in stressful situations.

**Day 4: Raising emotional awareness** [inspired by Greenberg (2002) and Halamová and Kanovský (2019)]

The goal of this exercise is for participants to learn to identify more precisely what they feel in any given situation (through becoming aware of situations, triggers, thoughts, body sensations, needs, desired behaviour, actual behaviour, and by naming their felt emotions).

**Day 5: Distinguishing between primary and secondary emotions and between adaptive and maladaptive emotions** [inspired by Halamová (2013) and Halamová and Kanovský (2019)]

Through this exercise, participants will learn to distinguish between primary and secondary emotions as well as between adaptive and maladaptive emotions. Participants are guided through choosing the most intense emotion and answer questions relating to the emotion.

**Day 6: Focusing** [inspired by Gendlin (1996), Halamová and Kanovský (2019), and McGuire (2007)].

This exercise involves creating a felt sense and using it to better understand inner experiences or to solve problems.

**Day 7: Emotion expression** [inspired by Ekman (2012), Greenberg (2002), and Halamová and Kanovský (2019)].

These exercises involve recalling a recent situation in which participants felt intense anger. After that participants are instructed to let the emerging feelings grow as strong as possible and to observe the changes to their body and face. Afterwards they are asked to describe the verbal and nonverbal signs of the emotion and compare their own expressions of anger with scientific findings about how anger is usually expressed. The same exercise is then performed with the emotion joy.

**Day 8: Articulating the relationship needs behind primary emotions** [inspired by Halamová (2013), Halamová and Kanovský (2019), and Johnson (2011)].

This exercise helps participants to identify the needs behind their emotions in a selected situation and what they need in the given situation and to articulate it.

**Day 9: Compliments** [inspired by Halamová (2013)].

This exercise teaches participants to give compliments to loved ones. Participants are instructed to think about the emotion they feel and then make a list of things that they appreciate about their loved ones and then compliment them. The compliment should be specific and relate to appearance, qualities, behaviour, skills, experience, relationships, values, performance, personality, character, etc.

**Day 10: Complaints** [inspired by the Gottman Institute (2011) and Halamová (2013)].

Based on research by the Gottman Institute (2011), this exercise teaches participants the skill of complaining constructively. The exercise involves learning a structure for formulating complaints and then practicing applying that structure to a series of past regrets.

**Day 11: Apology** [inspired by Dolhanty (2021) and Halamová and Kanovský (2019)].

During this exercise, participants are coached on how to apologize: what they need to say to somebody when they regret not having said something at the time the regrettable event happened.

**Day 12: Empathetic understanding** [inspired by Dolhanty (2021) and Halamová (2013)].

The exercise teaches participants how to be empathetic when talking to other people. The exercise involves learning a structure for formulating empathetic responses and then practicing applying that structure to a series of past relationship situations.

**Day 13: Facilitating emotional change** [inspired by Halamová (2013) and Pascual-Leone (2017)].

Participants are guided toward remembering a situation from the recent past that is emotionally discomforting. They are instructed to answer several questions about their emotional state and the exercise helps them facilitate an emotional change in the situation.

**Day 14: Self-care** [inspired by Ellison and Greenberg (2007) and Halamová (2013)].

At the end of an intervention, it is usually essential that participants are able to cope with a period of higher stress levels. It helps to prepare thoroughly for such periods in advance (Halamová et al., 2019). This exercise helps participants to cope with difficult moments and overcome the feelings of elevated vulnerability that often accompany stressful situations.

**Day 15–18: after-intervention measures.**

Post-measurements.

### Data analyses

The data analysis was performed using IBM SPSS statistics program version 27 (IBM Corporation, 2020). Cronbach alpha was calculated to assess the reliability of each scale and subscale. After that, we deployed the Shapiro-Wilks Normality test to calculate the data distribution. See the results in Appendix 1. Where the data distribution was not violated, we used paired t-Tests and calculated Cohen’s d for effect size results. Where the data distribution was not normal, we deployed the Wilcoxon signed-rank test and the Mann–Whitney U test to calculate the effect size.

## Results

The reliability analysis of the exploited scales and their subscales showed the following results: for FSCRS IS 0.877; RS 0.824; HS 0.700; IS+HS 0.885, for SOCS 0.856; RS 0.814; US 0.768; FS 0.687; TS 0.759; MA 0.712, for s-SIB distress 0.932; NA 0.756; PL 0.762; IA 0.694; PA 0.761, and for s-SIB performance 0.907; FNA 0.715; FPL 0.753; FIA 0.627; FPA 0.676. All were at an acceptable level.

### Self-compassion

A statistically significant effect of medium strength was obtained for the total score of self-compassion, *t* = −3,358; *df* = 82; *p* = 0,001; *d* = 0.52, and for the subscales (FS) feeling for the person suffering *Z* = −3,203; *p* = 0,001; *r_m_* = 0.35 and (MA) motivation to act/acting to alleviate suffering *Z* = −3,063; *p* = 0,002; *r_m_* = 0.34. For the (RS) recognizing suffering subscale there was a statistically significant effect of small strength *Z* = −2,369; *p* = 0,018; *r_m_* = 0.26. There was no statistically significant difference between the pre-test and post-test scores for (US) understanding the universality of suffering (*Z* = −0,702; *p* = 0,482; *r_m_* = 0.03) and (TS) tolerating uncomfortable feelings (*t* = −1,964; *df* = 82; *p* = 0,053; *d* = 0.31).

### Self-criticism

We found a statistically significant and medium size effect for self-criticism (IS+HS) in the pre-test and post-test measurements, *t* = 3,569; *df* = 82; *p* = 0,001; *d* = 0.55; and for the subscales (IS) Inadequate Self, *t* = 3,420; *df* = 82; *p* = 0,000; *d* = 0.61 and (RS) Reassured Self, *Z* = −4,970; *p* = 0,000; *r_m_* = 0.55. There was no statistically significant difference between the pre-test and post-test scores for the Hated Self subscale (*Z* = −1,788; *p* = 0,074; *r_m_* = 0.20).

### Self-protection/assertiveness

We did not find a statistically significant difference between the pre-test and post-test measurements for the total assertiveness score (*t* = 1,444; *df* = 82; *p* = 0,153; *d* = 0.23), or any of the s-SIB distress dimensions (NA) Negative Assertions (*t* = 0,568; *df* = 82; *p* = 0,572; *d* = 0.09), (PL) Expression of and Dealing with Personal Limitations (*Z* = −1,418; *p* = 0,156; *r_m_* = 0.16), (IA) Initiating Assertiveness (*t* = 3,420; *df* = 82; *p* = 0,081; *d* = 0.53), and (PA) Positive Assertions (*t* = 1,227; *df* = 82; *p* = 0,223; *d* = 0.19).

Similarly, there was no significant difference in the total s-SIB performance score (*t* = 0,025; *df* = 82; *p* = 0,980; *d* = 0.00), or any of its subscales (FNA) Negative Assertions (*t* = −0,428; *df* = 82; *p* = 0,670; *d* = 0.07), (FPL) Expression of and Dealing with Personal Limitations (*t* = 0,022; *df* = 82; *p* = 0,982; *d* = 0.00), (FIA) Initiating Assertiveness (*t* = −0,432; *df* = 82; *p* = 0,667; *d* = 0.07), and (FPA) Positive Assertions (*t* = 0,894; *df* = 82; *p* = 0,374; *d* = 0.14).

## Discussion

The aim of the research study was to develop a mobile app based on the original group training called Emotion-Focused Training for Emotional Coaching (EFT-EC; Halamová and Kanovský, 2019) and to test its effectiveness in reducing self-criticism and raising self-compassion and self-protection. Based on the results, we found support for its effectiveness in relation to self-criticism and self-compassion, but not self-protection.

We found a statistically significant effect for the total self-compassion score (SOCS-S) as well its subscales (FS) feeling for the person suffering, (MA) motivation to act/acting to alleviate suffering, and (RS) recognizing suffering. The app focused on learning emotional skills so it is understandable that participants were better able to recognize suffering and feel more for the suffering person, which could lead to enhanced motivation to help. The EFT-EC intervention incorporated questions about cognitions, emotions, and behaviours and probed participants to reflect on these three aspects of life. This could have heightened the positive effect of the intervention on participants as they may have processed the knowledge gained more effectively. No effect was found for the dimensions of (US) understanding the universality of suffering and (TS) tolerating uncomfortable feelings as the app did not deal with universality. The focus was more on emotional awareness rather than directly tolerating feelings.

Our results revealed a positive effect on the total score for self-criticism and the subscales (IS) Inadequate Self and (RS) Reassured Self. No effect was found in the hated-self dimensions but that is not surprising given its clinical nature and the more severe symptoms of the population scoring high on this dimension (Gilbert et al., 2004). Both self-criticism and self-compassion affect individuals greatly. Self-compassionate individuals are happier (Zessin et al., 2015) and better able to cope with stress and anxiety (Neff et al., 2007). Our results suggest that learning emotional skills could improve emotional skills but also self-compassion and self-criticism, which could yield greater happiness and better coping skills.

We have confirmed the previous empirical finding (Heffernan et al., 2010; Kemeny et al., 2012) that individuals can cultivate self-compassion by learning emotional skills. Even though just two of the tasks in the intervention focused directly on self-compassion, the intervention mediated the unforgiving side of self-critical thoughts (Gilbert and Irons, 2005). We also found that use of the short mobile intervention could enhance emotional intelligence, exert a direct positive effect on self-compassion (Heffernan et al., 2010), and possibly has an effect on emotional resilience as well (Beaumont et al., 2016). Empirical findings suggest that cultivating the protective role of self-compassion can reduce chronic stress and its impact on emotional responses (Lazarus and Folkman, 1984; Neff and Vonk, 2009) and transform negative emotional states (Carver and Connor-Smith, 2010). We found a similar effect, as the use of self-compassion is a beneficial emotional regulation strategy (Neff, 2003) that signifies emotional maturity (Maslow, 1997) and could be learned by leveraging low-cost, accessible online intervention delivered through a mobile app. Similarly to the findings of Kemeny et al. (2012) we found that by cultivating emotional balance we were able to foster self-compassion capabilities (Kemeny et al., 2012; Sansó et al., 2017).

We were able to successfully incorporate Emotion Focus Therapy methods into an accessible online intervention that had a positive effect on self-compassion and self-criticism. However, it did not have a significant effect on self-protection or assertiveness. This could be explained by the absence of direct exercises on self-protection. Only two tasks – Complaints and, to some extent, Articulating the Relationship Needs Behind Primary Emotions – were related to self-protection. Specific interventions with more assertiveness and self-protection tasks could perhaps obtain more significant results in this dimension, as healthy self-protection has wide-ranging benefits. In order to effectively raise self-protection, the EFT-EC mobile app should include exercises for developing these skills. A lack of self-protective and assertive behaviours may result in submissiveness, excessive aggression, negative emotions (Speed et al., 2018), or even depressive symptoms, social anxiety, and affect life satisfaction (Peneva and Mavrodiev, 2013; Speed et al., 2018).

Our research study did not confirm that learning emotional intelligence skills is not closely related assertiveness or self-protection skills; although some scholars propose that assertiveness is part of emotional intelligence [see Bar-On (1997)].

Traditionally, self-compassion and self-criticism interventions have been delivered in face-to-face group therapy settings (e.g., CFT, Gilbert, 2009; CEB, Kemeny et al., 2012). This creates an effective, but time-consuming and expensive solution to the problem (Chandrashekar, 2018). Like previous studies (Finlay-Jones et al., 2016; Eriksson et al., 2018; Mak et al., 2018; Linardon et al., 2019) we found that it is possible to increase self-compassion and reduce self-criticism by offering short online mobile apps or online-based apps. These are a cost-effective easy-to-administer solution that has a positive effect by raising self-compassion, mindfulness, and well-being and by reducing depression, anxiety, and psychological distress (MacBeth and Gumley, 2012; Kirby et al., 2017). Some people prefer online interventions as there are barriers to traditional therapy sessions (cost, negative stigma, unavailability, lack of anonymity; Wahbeh et al., 2014). Nonetheless, online interventions have many drawbacks, such as data safety and the absence of empirical research, and lack the personal touch that is reminiscent of human interaction (Anthes, 2016; Bakker et al., 2016).

## Limitations

The study design did not include a control group and so we could not statistically assess the effect by comparing active and passive participants. Therefore, there is a possibility that the changes could be attributed to the time and not the intervention itself. In addition, the mobile app was tested in one particular language and culture. It would be good to translate the app and see how it works in different languages. Finally, the participants were self-selected and there was no feedback so we can only speculate as to the reason for the premature dropout rate.

## Future studies

Future research could focus on effectiveness of this intervention and/or app design in different cultures or with subjects speaking different languages. Currently, we are in the process of gathering data from culturally non-specific English-speaking participants. In addition, we suggest measurement of emotion intelligence in future so it is tested whether the intervention significantly increase the ability to process emotions which is the main target of the intervention based on its name and the content.

## Conclusion

The 14-day mobile app based on the empirically supported Emotion Focused Training for Emotion Couching (EFT-EC) significantly improved self-compassion and self-reassurance, and significantly reduced self-criticism, comparing pre-and post-measurements. The results are promising as reducing self-criticism, a transdiagnostic phenomenon of various kinds of psychopathology, could prevent the emergence of psychopathologies. Furthermore, the mobile app intervention could be made easily accessible to a wide range of users without having to involve a mental health professional and hence without the potential risk of shame or stigmatization.

## Data availability statement

The raw data supporting the conclusions of this article will be made available by the authors, without undue reservation.

## Ethics statement

The studies involving human participants were reviewed and approved by The Ethical Committee of Faculty of Social and Economic Sciences, Comenius University in Bratislava. The patients/participants provided their written informed consent to participate in this study.

## Author contributions

JH designed the intervention and JM performed the statistical analysis. JH and JM designed research. JM, JH, and LB collected data. All authors wrote the first draft of the article, interpreted the results, revised the manuscript, and read and approved the final manuscript.

## Funding

This study was supported by the Vedecká grantová agentúra VEGA under Grant 1/0075/19 and the Slovak Research and Development Agency under the Contract no. PP-COVID-20-0074.

## Conflict of interest

The authors declare that the research was conducted in the absence of any commercial or financial relationships that could be construed as a potential conflict of interest.

## Publisher’s note

All claims expressed in this article are solely those of the authors and do not necessarily represent those of their affiliated organizations, or those of the publisher, the editors and the reviewers. Any product that may be evaluated in this article, or claim that may be made by its manufacturer, is not guaranteed or endorsed by the publisher.

## Figures and Tables

**Table 1:** 

	Shapiro-Wilkes Normality Test						Cronbach’s α
Scales and dimensions	Pretest			Postest			
	Statistic	Df	Sig.	Statistic	Df	Sig.	
FSCRS IS	0.974	84	0.088	0.98	84	0.217	0.877
FSCRS RS	0.944	84	0.085	0.965	84	0.023	0.824
FSCRS HS	0.944	84	0.001	0.931	84	0	0.7
FSCRS IS+HS	0.973	84	0.08	0.978	84	0.166	0.885
SOCS-S	0.978	84	0.574	0.98	84	0.221	0.856
SOCS-S RS	0.933	84	0	0.899	84	0	0.814
SOCS-S US	0.815	84	0	0.77	84	0	0.768
SOCS-S FS	0.963	84	0.016	0.972	84	0.065	0.687
SOCS-S TS	0.981	84	0.266	0.979	84	0.199	0.759
SOCS-S MA	0.974	84	0.092	0.958	84	0.009	0.712
s-SIB	0.978	84	0.162	0.978	84	0.17	0.932
s-SIB NA	0.986	84	0.491	0.983	84	0.327	0.756
s-SIB PL	0.978	84	0.172	0.969	84	0.041	0.762
s-SIB IA	0.983	84	0.33	0.989	84	0.723	0.694
s-SIB PA	0.982	84	0.279	0.961	84	0.013	0.761
s-SIB F	0.987	84	0.59	0.98	84	0.231	0.907
s-SIB FNA	0.983	84	0.333	0.983	84	0.335	0.715
s-SIB FPL	0.988	84	0.649	0.987	84	0.561	0.753
s-SIB FIA	0.974	84	0.088	0.98	84	0.225	0.627
s-SIB FPA	0.981	84	0.266	0.982	84	0.299	0.676

## Appendix 1

The results of Shapiro-Wilks Normality tests and Cronbach’s alpha for internal consistency.

## References

[ref2] Anthes E. (2016). Mental health: There’s an app for that. Nature 532, 20–23. doi: 10.1038/532020a, PMID: 27078548

[ref01] Arrindell W. A. Sanavio E. Sica C. (2002). Introducing a short form version of the Scale of Interpersonal Behaviour (s-SIB) for use in Italy. Psicoterapia Cognitiva e Comportamentale. 8, 3–18.

[ref3] Baião R. Gilbert P. McEwan K. Carvalho S. (2014). Forms of self-criticising/attacking & self-reassuring scale: psychometric properties and normative study. Psychol. Psychother. Theory Res. Pract. 88, 438–452. doi: 10.1111/papt.12049, PMID: 25492845

[ref4] Bakker D. Kazantzis N. Rickwood D. Rickard N. (2016). Mental health smartphone apps: review and evidence-based recommendations for future developments. JMIR Mental Health 3:e7. doi: 10.2196/mental.4984, PMID: 26932350 PMC4795320

[ref5] Bar-On R. (1997). The Emotional Intelligence Inventory (EQ-I). Technical Manual. Toronto: Multi-Health systems.

[ref6] Beaumont E. Irons C. Rayner G. Dagnall N. (2016). Does compassion-focused therapy training for health care educators and providers increase self-compassion and reduce self-persecution and self-criticism? J. Contin. Educ. Health Prof. 36, 4–10. doi: 10.1097/CEH.0000000000000023, PMID: 26954239

[ref7] Bergner R. M. (1995). Pathological Self-Criticism: Assessment and Treatment. New York: Plenum Press.

[ref8] Blatt S. J. (1974). Levels of object representation in anaclitic and introjective depression. Psychoanal. Study Child 29, 107–157. doi: 10.1080/00797308.1974.11822616, PMID: 4445397

[ref9] Blatt S. J. Zuroff D. C. (1992). Interpersonal relatedness and self-definition: two prototypes for depression. Clin. Psychol. Rev. 12, 527–562. doi: 10.1016/0272-7358(92)90070-o

[ref08] Carver C. S. Connor-Smith J. (2010). Personality and coping. Annu. Rev. Psychol. 61, 679–704. doi: 10.1146/annurev.psych.093008.100352 19572784

[ref11] Chandrashekar P. (2018). Do mental health mobile apps work: evidence and recommendations for designing high-efficacy mental health mobile apps. Mhealth 4:6. doi: 10.21037/mhealth.2018.03.02, PMID: 29682510 PMC5897664

[ref12] Corporation IBM. (2020). IBM SPSS statistics for windows, version 27.0. Armonk, NY: IBM Corp.

[ref13] Cox B. J. Walker J. R. Enns M. W. Karpinski D. C. (2002). Self-criticism in generalized social phobia and response to cognitive-behavioral treatment. Behav. Ther. 33, 479–491. doi: 10.1016/s0005-7894(02)80012-0

[ref14] Dolhanty J. (2021). Emotion focused skills training. Unpublished materials.

[ref15] Ekman P. (2012). Emotions revealed. New York: Holt Paperbacks.

[ref16] Ellison J. A. Greenberg L. S. (2007). “Emotion-focused experiential therapy” in Handbook of Homework Assignments in Psychotherapy. eds. Kazantzis N. L’Abate L. (New York, NY: Springer), 65–83.

[ref17] Eriksson T. Germundsjö L. Åström E. Rönnlund M. (2018). Mindful self-compassion training reduces stress and burnout symptoms among practicing psychologists: a randomized controlled trial of a brief web-based intervention. Front. Psychol. 9:2340. doi: 10.3389/fpsyg.2018.02340, PMID: 30538656 PMC6277494

[ref18] Ferrari M. Hunt C. Harrysunker A. Abbott M. J. Beath A. P. Einstein D. A. (2019). Self-compassion interventions and psychosocial outcomes: a meta-analysis of RCTS. Mindfulness 10, 1455–1473. doi: 10.1007/s12671-019-01134-6

[ref19] Finlay-Jones A. Kane R. Rees C. (2016). Self-compassion online: a pilot study of an internet-based self-compassion cultivation program for psychology trainees. J. Clin. Psychol. 73, 797–816. doi: 10.1002/jclp.22375, PMID: 27787877

[ref20] Gendlin E.T. (1996). Focusing Psychotherapy: A Manual of the Experiential Method. New York: Guilford.

[ref21] Gilbert P. (2009). The Compassionate Mind: A New Approach to the Challenges of Life. London: Constable & Robinson.

[ref22] Gilbert P. Clark M. Hempel S. Miles J. N. V. Irons C. (2004). Criticising and reassuring oneself: an exploration of forms, styles and reasons in female students. Br. J. Clin. Psychol. 43, 31–50. doi: 10.1348/014466504772812959, PMID: 15005905

[ref222] Gilbert P. Irons C. (2004). A pilot exploration of the use of compassionate images in a group of self-critical people. Memory 12, 507–516. 15487546 10.1080/09658210444000115

[ref23] Gilbert P. Irons C. (2005). “Compassionate mind training, for shame and self-attacking, using cognitive, behavioral, emotional and imagery interventions” in Compassion: Conceptualizations, Research, and Use in Psychotherapy. ed. Gilbert P. (London: Brunner-Routledge), 263–325. doi: 10.4324/9780203003459-15

[ref24] Gottman Institute (2011). How to complain without hurting your partner. Available at: http://www.youtube.com/watch?v=bShsyKUFjKE

[ref25] Gottman J. M. Silver N. (2000). The Seven Principles for Making Marriage Work: A Practical Guide from the Country's Foremost Relationship Expert. New York: Three Rivers Press.

[ref26] Greenberg L. (2002). Emotion-Focused Therapy: Coaching Clients to Work through Feelings. Washington, D.C.: American Psychological Association Press.

[ref27] Greenberg L. S. (2011). Emotion-Focused Therapy. Washington, DC: American Psychological Association.

[ref28] Greenberg L. S. Warwar S. H. (2006). Homework in an emotion-focused approach to experiential therapy. J. Psychother. Integr. 16, 178–200. doi: 10.1037/1053-0479.16.2.178

[ref29] Gu J. Baer R. Cavanagh K. Kuyken W. Strauss C. (2020). Development and psychometric properties of the Sussex-Oxford compassion scales (SOCS). Assessment 27, 3–20. doi: 10.1177/1073191119860911 31353931 PMC6906538

[ref30] Halamová J. (2013). Terapia zameraná na emócie I. Učebnica. Bratislava: Vydavateľstvo UK.

[ref31] Halamová J. Kanovský M. (2019). Emotion-focused training for emotion coaching - an intervention to reduce self-criticism. Hum. Aff. 29, 20–31. doi: 10.1515/humaff-2019-0003

[ref32] Halamová J. Kanovský M. (2021). Factor structure of the Sussex-Oxford Compassion Scales. Psihologijske Teme 30, 489–508. doi: 10.31820/pt.30.3.5

[ref33] Halamová J. Kanovský M. Pacúchová M. (2017). Robust psychometric analysis and factor structure of the Forms of Self–criticizing/Attacking and Self–reassuring Scale. Česk. Psychol. 61, 331–349.

[ref07] Halamová J. Kanovský M. Varšová K. Kupeli N. (2021). Randomised controlled trial of the new short-term online emotion focused train-ing for self-compassion and self-protection in a nonclinical sample. Curr. Psychol. 40, 333–343. doi: 10.1007/s12144-018-993 33488040 PMC7799374

[ref34] Halamová J. Koróniová J. Kanovský M. Kénesy Túniyová M. Kupeli N. (2019). Psychological and physiological effects of emotion focused training for self-compassion and self-protection. Res. Psychother. 22, 265–280. doi: 10.4081/ripppo.2019.358, PMID: 32913797 PMC7451316

[ref35] Heffernan M. Quinn M. T. McNulty R. Fitzpatrick J. J. (2010). Self compassion and emotional intelligence in nurses. Int. J. Nurs. Pract. 16, 366–373. doi: 10.1111/j.1440-172X.2010.01853.x 20649668

[ref36] Johnson S. M. (2011). Hold me tight. London: Piatkus.

[ref37] Kemeny M. E. Foltz C. Cavanagh J. F. Cullen M. Giese-Davis J. Jennings P.. (2012). Contemplative/emotion training reduces negative emotional behavior and promotes prosocial responses. Emotion 12, 338–350. doi: 10.1037/a0026118, PMID: 22148989

[ref38] Kirby J. N. Tellegen C. L. Steindl S. R. (2017). A meta-analysis of compassion-based interventions: current state of knowledge and future directions. Behav. Ther. 48, 778–792. doi: 10.1016/j.beth.2017.06.003, PMID: 29029675

[ref39] Krieger T. Martig D. S. van den Brink E. Berger T. (2016). Working on self-compassion online: a proof of concept and feasibility study. Internet Interv. 6, 64–70. doi: 10.1016/j.invent.2016.10.001, PMID: 30135815 PMC6096262

[ref40] Lazarus R. S. Folkman S. (1984). Stress, Appraisal, and Coping. New York, NY: Springer Publishing Company.

[ref41] Leaviss J. Uttley L. (2014). Psychotherapeutic benefits of compassion-focused therapy: an early systematic review. Psychol. Med. 45, 927–945. doi: 10.1017/s0033291714002141, PMID: 25215860 PMC4413786

[ref42] Linardon J. Cuijpers P. Carlbring P. Messer M. Fuller-Tyszkiewicz M. (2019). The efficacy of app-supported smartphone interventions for mental health problems: a meta-analysis of randomized controlled trials. World Psychiatry 18, 325–336. doi: 10.1002/wps.20673, PMID: 31496095 PMC6732686

[ref43] Linehan M.M. (1993). Skills Training Manual for Treating Borderline Personality Disorder. New York: Guilford Press.

[ref44] MacBeth A. Gumley A. (2012). Exploring compassion: a meta-analysis of the association between self-compassion and psychopathology. Clin. Psychol. Rev. 32, 545–552. doi: 10.1016/j.cpr.2012.06.003, PMID: 22796446

[ref45] Mak W. W. Tong A. C. Yip S. Y. Lui W. W. Chio F. H. Chan A. T.. (2018). Efficacy and moderation of mobile app–based programs for mindfulness-based training, self- compassion training, and cognitive behavioral psychoeducation on mental health: randomized controlled noninferiority trial. JMIR Mental Health 5:e60. doi: 10.2196/mental.8597 30309837 PMC6231823

[ref46] Maslow A. (1997). Toward a Psychology of Being, 2nd Edn. New York: Van Nostrand Reinhold.

[ref47] McGuire K. (2007). Complete focusing instructions. Available at: http://www.cefocusing.com/wordpress/?p=75

[ref48] Mellor D. Firth L. Moore K. (2008). Can the internet improve the well-being of the elderly? Ageing Int. 32, 25–42. doi: 10.1007/s12126-008-9006-3

[ref49] Neff K. D. (2003). Self-compassion: an alternative conceptualization of a healthy attitude toward oneself. Self Identity 2, 85–101. doi: 10.1080/15298860309032

[ref51] Neff K. D. Kirkpatrick K. L. Rude S. S. (2007). Self-compassion and adaptive psychological functioning. J. Res. Pers. 41, 139–154. doi: 10.1016/j.jrp.2006.03.004

[ref52] Neff K. D. Vonk R. (2009). Self-compassion versus global self-esteem: two different ways of relating to oneself. J. Pers. 77, 23–50. doi: 10.1111/j.1467-6494.2008.00537.x, PMID: 19076996

[ref53] O’Connor R. C. Noyce R. (2008). Personality and cognitive processes: self-criticism and different types of rumination as predictors of suicidal ideation. Behav. Res. Ther. 46, 392–401. doi: 10.1016/j.brat.2008.01.007, PMID: 18308293

[ref54] Orosa-Duarte Á. Mediavilla R. Muñoz-Sanjose A. Palao Á. Garde J. López-Herrero V.. (2021). Mindfulness-based mobile app reduces anxiety and increases self-compassion in healthcare students: a randomised controlled trial. Med. Teach. 43, 686–693. doi: 10.1080/0142159x.2021.1887835 33645416

[ref55] Pascual-Leone A. (2017). How clients “change emotion with emotion”: a programme of research on emotional processing. Psychother. Res. 28, 165–182. doi: 10.1080/10503307.2017.1349350 28714778

[ref56] Pascual-Leone A. Greenberg L. (2007). Emotional processing in experiential therapy: why “the only way out is through”. J. Consult. Clin. Psychol. 75, 875–887. doi: 10.1037/0022-006X.75.6.875 18085905

[ref57] Peneva I. Mavrodiev S. (2013). A historical approach to assertiveness. Psychol. Thought 6, 3–26. doi: 10.5964/psyct.v6i1.14

[ref03] Pennebaker J. W. (2017). Expressive Writing in Psychological Science. Perspect. Psychol. Sci. 13, 226–229. doi: 10.1177/1745691617707315 28992443

[ref04] Pennebaker J. W. Beall S. K. (1986). Confronting a traumatic event: Toward an understanding of inhibition and disease. J. Abnorm. Psychol. 95, 274–281. doi: 10.1037/0021-843x.95.3.274 3745650

[ref58] Sansó N. Galiana L. Cebolla A. Oliver A. Benito E. Ekman E. (2017). Cultivating emotional balance in professional caregivers: a pilot intervention. Mindfulness 8, 1319–1327. doi: 10.1007/s12671-017-0707-0

[ref59] Segal J. (2008). The Language of Emotional Intelligence: The Five Essential Tools for Building Powerful and Effective Relationships. New York: McGraw-Hill.

[ref60] Speed B. C. Goldstein B. L. Goldfried M. R. (2018). Assertiveness training: a forgotten evidence-based treatment. Clin. Psychol. Sci. Pract. 25, 1–20. doi: 10.1111/cpsp.12216

[ref61] Statista. (2021). Forecast number of mobile users worldwide 2020–2025. Statista. Available at: https://www.statista.com/statistics/218984/number-of-global-mobile-users-since-2010/ (Accessed March 12, 2022).

[ref09] Stein S. J. Book H. E. (2006). The EQ edge: emotional intelligence and your success, (2nd edition). Toronto, Canada: Multi-Health Systems.

[ref02] Strauss C. Taylor B. L. Gu J. Kuyken W. Baer R. Jones F.. (2016). What is compassion and how can we measure it? A review of definitions and measures. Clin. Psychol. Rev. 47, 15–27. doi: 10.1016/j.cpr.2016.05.004 27267346

[ref06] Timulak L. (2015). Transforming Emotional Pain in Psychotherapy: An Emotion-Focused Approach. London, UK: Routledge, 183.

[ref62] Torous J. Lipschitz J. Ng M. Firth J. (2020). Dropout rates in clinical trials of smartphone apps for depressive symptoms: a systematic review and meta-analysis. J. Affect. Disord. 263, 413–419. doi: 10.1016/j.jad.2019.11.167, PMID: 31969272

[ref1] Vráblová V. Halamová J. (2022). Short Version of the Scale for Interpersonal Behavior (s-SIB): Slovak Translation, Psychometric Analysis, and Factor Structure. Front. Psychol. 13:1024530. doi: 10.3389/fpsyg.2022.1024530 36582340 PMC9792610

[ref05] Vrana G. Greenberg L. (2018). Overview of emotion‐focused therapy. ed. Foroughe M.. Emotion focused family therapy with children andcaregivers: A trauma‐informed approach. New York, NY: Routledge, 1–22.

[ref63] Wahbeh H. Svalina M. N. Oken B. S. (2014). Group, one-on-one, or internet? Preferences for mindfulness meditation delivery format and their predictors. Open Med. J. 1, 66–74. doi: 10.2174/1874220301401010066, PMID: 27057260 PMC4820831

[ref64] Zessin U. Dickhauser O. Garbade S. (2015). The relationship between self-compassion and well-being: a meta-analysis. Appl. Psychol. Health Well Being 7, 340–364. doi: 10.1111/aphw.12051, PMID: 26311196

